# A Meta-Analysis of Studies Evaluating Visual and Anatomical Outcomes in Patients with Treatment Resistant Neovascular Age-Related Macular Degeneration following Switching to Treatment with Aflibercept

**DOI:** 10.1155/2016/4095852

**Published:** 2016-03-06

**Authors:** Sophie Seguin-Greenstein, Sue Lightman, Oren Tomkins-Netzer

**Affiliations:** ^1^Moorfields Eye Hospital, 162 City Road, London EC1V 2PD, UK; ^2^UCL Institute of Ophthalmology, 11-43 Bath Street, London EC1V 9EL, UK; ^3^Royal Surrey County Hospital, Egerton Road, Guildford, Surrey GU2 7XX, UK; ^4^Bnai Zion Medical Center, 47 Golomb Road, 31048 Haifa, Israel

## Abstract

With the introduction of aflibercept, eyes with neovascular age-related macular degeneration (AMD) not responding well to injections of ranibizumab or bevacizumab can be switched to treatment with aflibercept. We carried out a meta-analysis to analyze all available evidence of visual and anatomical outcomes of eyes with resistant neovascular AMD switched to aflibercept at six months. Data from seven retrospective and prospective studies looking at change in best corrected visual acuity (BCVA) and central retinal thickness (CRT) were included. Weighted mean difference (WMD) and 95% CI were estimated using the standardized mean change method. The overall results of the meta-analysis showed a small but statistically significant improvement in BCVA six months following treatment switch to aflibercept (WMD 0.142, 95% CI 0.006 to 0.28; *p* = 0.04), and the effect was more significant in data gathered from prospective studies (WMD 0.407, 95% CI 0.023 to 0.791, *p* = 0.038). There was a significant improvement in CRT following treatment switch to aflibercept (WMD −0.36, 95% CI −0.485 to −0.235; *p* < 0.0001). Our meta-analysis indicates that following treatment switch to aflibercept patients may have a significant improvement in CRT with stabilization or even some improvement in their visual acuity.

## 1. Introduction

Age-related macular degeneration (AMD) is a chronic degenerative process and is the leading cause of severe vision loss in people over the age of 60 in developed countries [1]. The neovascular (wet or exudative) form of AMD, which accounts for approximately 10% of cases, results in rapid deterioration in visual acuity often with permanent severe loss of vision [2]. The identification of the pathophysiologic mechanisms at the basis of neovascular AMD, particularly the role of vascular endothelial growth factor (VEGF), has led to the development and use of intravitreally delivered anti-VEGF agents, which target and cause regression of choroidal neovascularization [3, 4] and have become the standard of care. Currently, there are three clinically available agents, ranibizumab (Lucentis, Genentech, South San Francisco, CA), bevacizumab (Avastin, Genentech), and a more recent addition aflibercept (Eylea, Regeneron, Tarrytown, NY, USA). Each of these drugs has been tested in large multicenter, randomized, controlled clinical trials and found to have comparable effects on treatment of naïve patients [5–8]. To date, no clear evidence has been presented demonstrating a significant difference between aflibercept and any other agent for the treatment of either naïve or resistant neovascular AMD. Indeed, patients that are deemed unresponsive to treatment with any one agent are regularly offered an alternative drug. Treatment protocols with aflibercept also differ among studies, with a loading dose of three monthly injections followed either by a bimonthly or by a pro re nata regimen. Recently, a study examining the response to anti-VEGF treatment, using all three drugs, of patients with diabetic macular edema demonstrated that eyes with severe vision loss were more likely to benefit from the use of aflibercept [9]. This suggests that there may be a difference in drug response among some patient subgroups.

In this paper we aimed to perform a meta-analysis of the published literature on the efficacy of aflibercept in patients with neovascular AMD resistant to previous treatment with ranibizumab and/or bevacizumab. We evaluated the changes in their visual acuity and central retinal thickness (CRT) following the switch to aflibercept.

## 2. Methods

### 2.1. Search Strategy

Major databases including PubMed (MEDLINE), EMBASE, the Science Citation Index Expanded, and the Cochrane Central Register of Controlled Trials (CENTRAL) in the Cochrane Library were searched for studies comparing visual acuity and/or change in CRT on optical coherence tomography (OCT) of eyes with resistant neovascular AMD on prior anti-VEGF treatment switching to aflibercept, published in English from January 2012 to May 2015. The medical search headings “ranibizumab,” “bevacizumab,” “Avastin,” “anti-VEGF,” “Lucentis,” “Aflibercept,” “Eylea,” and combinations of these were used, so were the keywords “persistent,” “resistant,” and “recurrent” and the keywords “switching,” “transitioning,” and “conversion.” The reference lists of articles identified were examined to find additional relevant studies that had not been identified by the database searches. We only included comparative clinical studies with the same group of patients treated with anti-VEGF prior to switching to aflibercept and that had full text available in English. The final inclusion of articles was determined by consensus between the authors SSG and OTN.

We followed the Preferred Reporting Items for Systematic Reviews and Meta-Analysis (PRISMA) guidelines in designing, performing, and reporting the systematic review [10]. Included studies were required to (1) assess visual acuity and/or CRT of patients with persistent and resistant neovascular AMD treated with one anti-VEGF drug and then switched to aflibercept, (2) have a minimum follow-up of six months after switching to aflibercept, (3) have the treatment regimen used clearly stated, and (4) be published in English.

### 2.2. Data Extraction and Quality Assessment

Data were extracted using standardized forms. Data recorded included patient and study characteristics, BCVA, CRT, and statistics used for the study. In order to evaluate the reliability of the comparative evidence, two authors (SSG and OTN) independently assessed the risk of bias of the included studies using a modified version of the Newcastle-Ottawa Scale for assessing the quality of prospective and retrospective studies in meta-analysis [11, 12]. Each paper was awarded a score in four categories, patient selection (0–3), treatment comparability (0–6), statistical methods (0–3), and outcome (0–6). Studies achieving ten or more points were considered to be of high quality. Only these studies were included in the final analysis. Prospective studies scored higher on patient selection than retrospective studies as were multicenter studies, as they are more likely to be representative of the entire patient population. Use of fluorescein fundus angiography (FFA), OCT, or combination of both received an increasingly higher score on treatment comparability. Outcome and timing assessments of best corrected visual acuity (BCVA) and CRT as well as reporting of follow-up were all evaluated. For the purpose of the analysis, all BCVA values were converted to the log minimum angle of resolution (LogMAR).

### 2.3. Statistical Analysis

Original data were extracted from the studies and analyzed and the standardized mean change was used to calculate intervention effects. The standardized mean change was used to compute the estimates of treatment for correlated designs. The standardized mean change has been shown to be a more appropriate measure of effect size for the direct comparison of data from studies using a pretest and posttest design without control groups [13–15]. For the purposes of this meta-analysis, we used the mean, standard deviation (SD), and the correlation for pre/postswitching data. In all studies, the correlation between pre- and postswitching data was not reported and values were obtained either directly from the authors or by calculating the correlation using the *p* value/*t*-test values, means, standard deviation, and the number of patients included from the published data [14]. Using these values we were then able to calculate the standardized mean change for each group [15]. Studies for which such information could not be obtained were excluded from the meta-analysis (*n* = 5). The standardized mean change was calculated as the difference between the means of the posttreatment switching values and the baseline divided by the pooled standard deviation. Five studies lacked enough statistical information to be included in this meta-analysis and their outcomes were reviewed for information only [16–20]. Studies specific standardized mean change was pooled using fixed effect models with the Mantel-Haenszel method if heterogeneity was negligible or using random effect models with the DerSimonian-Laird method when heterogeneity was significant [21, 22]. Interstudies heterogeneity was assessed using the Cochran *Q* test and *I* ^2^ tests [22], with a *p* < 0.05 and *I* ^2^ > 50% suggesting a high interstudy heterogeneity. In an attempt to identify the source of heterogeneity in the data, we performed a metaregression analysis on confounders such as the type of study, the previous total length of treatment, previous total number of anti-VEGF injections prior to switching, and mean BCVA at baseline. A *p* value < 0.05 was regarded as significant results and all tests were 2 sided. All statistical analyses were performed using commercially available software comprehensive meta-analysis (CMA, Biostat, Englewood, NJ, USA, v 3.0). The estimates of treatment effect are presented as weighted mean difference (WMD) and graphically as forest plots. Sensitivity analysis was carried out by including only studies deemed with a quality score of 90% or above (13 points and above) and excluding each study from the analysis of each outcome measure to confirm the stability of our findings. Publication bias was assessed with the funnel plot and with the Egger test [23].

## 3. Results

### 3.1. Search Findings and Results Characteristics

**Figure 1:**
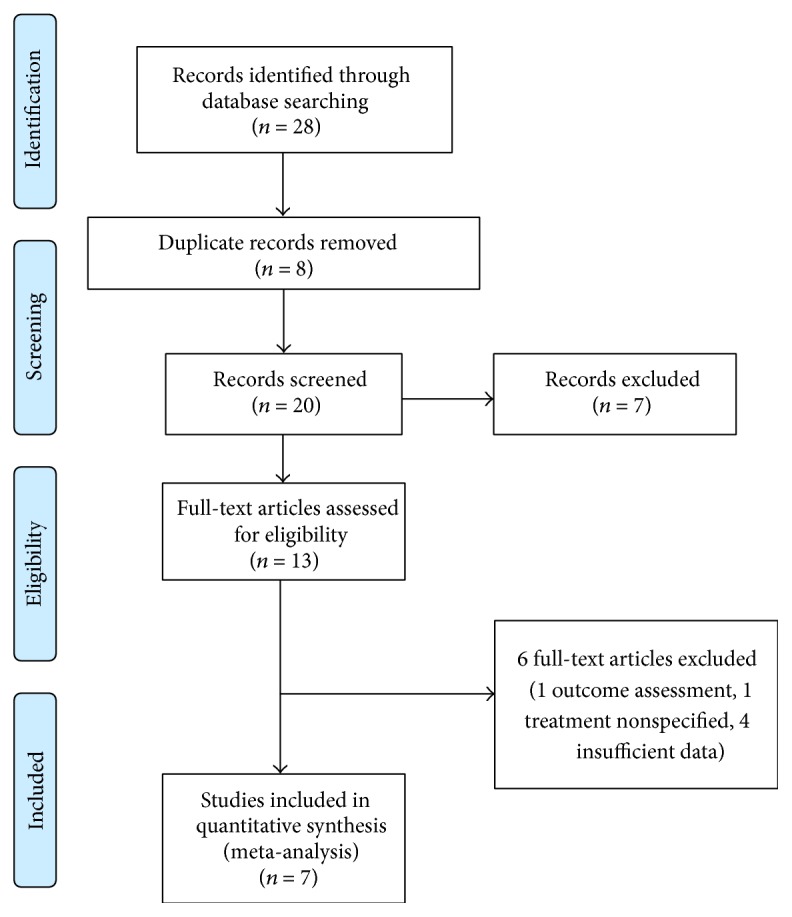
PRISMA flow diagram of study selection.

The search strategy initially generated 28 relevant clinical studies, of which 13 scored higher than 10 on the quality assessment. Of these, seven studies had sufficient outcome data and statistical information to be included in the meta-analysis (four retrospective studies [24–27] and 3 prospective studies [28–30], Figure 1). All these studies were included in the meta-analysis of visual and/or CRT following switching to aflibercept, six studies in the analysis of BCVA (232 eyes of 225 patients) [24–26, 28–30] and five in the analysis of CRT (266 eyes of 259 patients) [24–28].  Table 1 details study characteristics, quality, and comparability assessments, and Table 2 shows the analyzed outcome measures. Median patient age at time of treatment switching was 78 years (range 70.1–80.3 years), with a median female percentage of 55.96% (range 30.9–70.5%). At the time of treatment switching patients had been diagnosed with neovascular AMD for a median of 40.65 months (range 20.5–44.1 months) and eyes had already been given a median of 28.6 anti-VEGF injections (range 9.6–42.0 injections). The median time between the last anti-VEGF injection and beginning of aflibercept treatment was 35.0 days (range 33.3–46.5 days) and eyes were treated with a median of 5.1 aflibercept injections (range 4.50–5.60 injections). The median BCVA at baseline was 0.53 LogMAR (range 0.32–0.64 LogMAR) and CRT on OCT was 413 *µ*m (range 336.3–448.3 *µ*m).

### 3.2. Best Corrected Visual Acuity (6 Studies)

**Figure 2:**
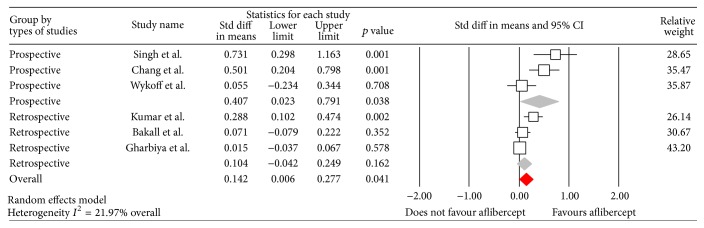
Forrest plot of six studies reporting comparative results of best corrected visual acuity six months after switching to aflibercept. Note that for the prospective studies the accumulated effect demonstrated a significant improvement, which was not found for the retrospective studies. The overall effect was found to be significant (*p* = 0.04). The lower and upper limits represent the 95% confidence interval.

The overall results of the meta-analysis showed a small but statistically significant improvement in BCVA at six months after switching to aflibercept (WMD 0.142, 95% CI 0.006 to 0.28; *p* = 0.04). The random model was used as heterogeneity of the data (*I* ^2^) was 81.02%. Exploring possible confounders on BCVA we performed a weighted regression on the six studies included. The type of study design was the only moderator that showed a significant effect on the BCVA at six months (*Q* = 8.71, *p* = 0.003), with prospective studies demonstrating a greater estimate of treatment effect on change in BCVA (WMD 0.407, 95% CI 0.02 to 0.79, *p* < 0.038, Figure 2) than retrospective studies (WMD 0.104, 95% CI −0.04 to 0.25; *p* = 0.16). Other covariates such as mean number of anti-VEGF injections prior to switching to aflibercept, length of time of treatment with anti-VEGF prior to switching, mean BCVA at switching, and the type of treatment had no effect on the overall results.

### 3.3. Central Retinal Thickness (5 Studies)

**Figure 3:**
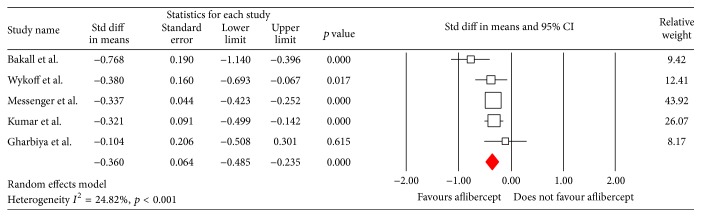
Forrest plot of five studies reporting comparative results of the central retinal thickness (CRT) six months after switching to aflibercept. The overall effect demonstrated a significant reduction in CRT. The lower and upper limit represents the 95% CI.

Overall, patients with their current treatment resistant AMD had a significant improvement in CRT at six months following switching to aflibercept (WMD −0.36, 95% CI −0.49 to −0.24; *p* <0.0001, Figure 3). The random model was used as heterogeneity of the data (*I* ^2^) was 66.79%. The weighted regression on the 5 studies included for covariates (mean number of anti-VEGF prior to switching to aflibercept, total length of treatment with anti-VEGF prior to switching, mean BCVA at switching, and mean CRT at switching) showed no significant effect on CRT at 6 months.

### 3.4. Sensitivity and Subgroup Analysis

We performed a sensitivity analysis, to determine the effect of each study on the overall result, by removing each of the studies one at a time and recalculating the summary WMD. The overall pooled WMD remained stable indicating that our results were not influenced by any single study. We performed a further sensitivity analysis of all studies scoring above 90% of the maximal score (13 points and above, WMD 0.204, CI from 0.012 to 0.395, *p* = 0.037) and excluding each study from the analysis of each outcome measure [26, 28–30]. These exclusions did not alter the results obtained in the overall analysis.

### 3.5. Publication Bias

We assessed possible publication bias with a funnel plot (not shown). Although the funnel plot showed evidence of publication bias, the small number of studies limits this analysis, as confirmed by Egger's test (*p* = 0.03). The same tests did not suggest publication bias for the anatomical outcome (*p* = 0.43).

## 4. Discussion

This study systematically reviewed and analyzed the evidence in the literature of the effect of switching to treatment with aflibercept in eyes with neovascular AMD resistant to previous anti-VEGF treatment. Overall, we included four retrospective and three prospective studies that had a follow-up period of at least six months (three retrospective studies had a follow-up period of up to twelve months). While we were able to identify other studies that examined the effect of switching to aflibercept, insufficient outcome information prevented these from being included in this analysis.

Our meta-analysis demonstrated a small but significant improvement in BCVA following switching to aflibercept. While this improvement in BCVA was indeed small and of limited clinical significance, it was driven by a significant improvement noted in the analyzed prospective studies (ranging between 0.1 and 0.14 LogMAR), suggesting that the retrospective design of the studies where no effect was found may have influenced their outcome.

The metaregression analysis did not demonstrate any effects on BCVA from confounders such as mean number of anti-VEGF injections, mean length of treatment or the drug used prior to switching, which is in keeping with results from other studies [19, 27]. While most studies used a standard treatment protocol following switching, of a loading dose of three monthly injections followed by a bimonthly regime, other studies used a pro re nata regimen, neither of which resulted in a difference in treatment effect.

The results of this meta-analysis demonstrated a significant improvement in CRT following the treatment switch to aflibercept. This was a consistent finding in all the studies and was maintained during the longer follow-up up to twelve months [27, 31, 32]. While there was a definite improvement in retinal thickness, this did not correlate with significant restoration of visual function. Repeated use of any drug can result over time in tachyphylaxis with resultant loss of clinical effect [33–35], which can often be overcome by switching to another drug with an alternative mode of action. Thus, restoration of effect may be achieved following switching, resulting in clearing of retinal fluid and reduction in CRT. However, the lack of a concomitant gain in visual function suggests it may be related either to an accumulating effect of long term neovascularization, such as development of retinal gliosis, or to progression of retinal atrophic changes that have been documented to occur in neovascular AMD eyes treated with anti-VEGF injections [36–38].

While this meta-analysis included all current studies examining treatment switching to aflibercept in eyes with neovascular AMD, it nevertheless suffers from several limitations, mainly related to the quality of the studies analyzed. Most studies performed to date were nonrandomized retrospective treatment comparison studies, which introduce potential confounding biases due to an inability to adjust for patient baseline demographic characteristics or disease severity. Though we found a significant improvement in CRT on OCT and BCVA, study design and population varied across studies and this variation was reflected in significant heterogeneity in the estimated comparative effect for the analysis of both BCVA and CRT. We found that the type of study had a significant impact on our results when analyzing BCVA, supporting the view that information gathered from prospective studies may offer clearer conclusions and that overinterpretation of retrospective studies should be avoided. Sensitivity and subgroup analysis of only high quality studies did not reveal any difference in the results and suggests that these results indeed represent the culmination of all current studies. The main strength of our meta-analysis is in incorporating all previous studies, resulting in a large sample size and allowing us to focus the results of all previous studies.

## 5. Conclusions

To date, this is the first systematic meta-analysis evaluating the visual and anatomical outcomes of patients with resistant AMD converted to aflibercept. Our analysis provides substantial evidence that following switching there is a significant anatomical effect, resulting in CRT thinning. However, the visual function change was far more modest and while there is evidence to support that aflibercept has a comparable effect to other anti-VEGF agents in maintaining vision, any potential significant benefit should be regarded with caution. While this study has clarified the known effect of aflibercept in treatment failure neovascular AMD eyes, future results, especially from prospective studies, may offer new insights into the different effects of these agents.

## Figures and Tables

**Table 1:** Characteristics of all studies included in the meta-analysis.

Authors	Year	Country	Study design	*N* (eyes)	Inclusion/exclusion criteria	Matching/comparable factors	Study quality (points scoring scale)
Kumar [24]	2013	USA	Retro	34	IRF, SRF, or sub-RPE with adjacent IRF/SRF on OCT	*BCVA, CRT*	Selection: 2 Comparability: 2 Stats:1 Outcome: 6 Overall:11

Bakall [25]	2013	USA	Retro	36	CNV confirmed by OCT and FFA at baseline visit IRF/SRF present at least for 3 months prior to conversion and treated with 3 monthly anti-VEGF injections	*BCVA, CRT *	Selection: 3 Comparability: 3 Stats: 1 Outcome: 4 Overall: **11**

Gharbiya [26]	2014	Italy	Retro	31	IRF/SRF on OCT At least 6 previous anti-VEGF injections Less than 4 weeks between last anti-VEGF treatment and conversion	*BCVA, CRT *	Selection: 2 Comparability: 2 Stats: 3 Outcome: 6 Overall: **13**

Messenger [27]	2014	USA	Retro	109	IRF, SRF, or sub-RPE with adjacent IRF/SRF on OCT At least 12 months of anti-VEGF treatment prior to conversion VA > 20/400 at conversion At least 12 months of follow-up	*CRT *	Selection: 2 Comparability: 3 Stats: 2 Outcome: 4 Overall: **11**

Wykoff [28]	2014	USA	Prosp	46	Patients who completed the 2-year SAVE trial	*BCVA,CRT *	Selection: 3 Comparability: 3 Stats: 3 Outcome: 6 Overall: **15**

Chang [29]	2014	Australia	Prosp	50	CNV on OCT and FFA At least 4 anti-VEGF injections prior to conversion	*BCVA *	Selection: 3 Comparability: 2 Stats: 3 Outcome: 6 Overall: **14**

Singh [30]	2014	USA	Prosp	26	Active CNV confirmed by FFA BCVA between 25 and 80 ETDRS letters at baseline At least one anti-VEGF injection within 3 months of conversion	*BCVA *	Selection: 3 Comparability: 2 Stats: 3 Outcome: 6 Overall: **14**

**Table 2:** Clinical characteristics of all studies included in the meta-analysis.

Authors	Mean age (years)	Duration of disease (months)	Nb of injections prior to conversion	Time between last anti-VEGF and conversion	Mean time of follow-up (months)	Mean number of aflibercept injections	Treatment regimen
Kumar [24]	79 (IQR 72–84)	44.7 (IQR 24–76)	28.6 (IQR 10–47)	34.4 days (IQR 32–37)	6	5.6 (NS)	Loading then PRN
Bakall [25]	79 (range 60–88)	NS	25.6 (6–74)	NS	6	5.2 (4–6)	Loading then PRN
Gharbiya [26]	70.1 (range 60–86)	41.3 (15–58)	34.4 (15–50)	5.1 weeks (range 4–6)	6	4.5 (3–6)	Loading then PRN
Messenger [27]	80.3 (range 59–96)	NS	21.4 (4–60)	NS	6, 12	7.2 (1–12) for 12 months NS at 6 months	Loading then PRN
Wykoff [28]	77.8 (range 55–95)	NS	42 (19–67)	33 days (range 28–68)	6	5.6 (4–6)	Loading then PRN
Chang [29]	77.8 (NS)	40	34.94	NS	6	NS	Loading then bimonthly
Singh [30]	78 (NS)	14	9.62 (3–23)	50 days (range 21–91)	6	NS	Loading then bimonthly
